# RETRACTED: Lobo et al. Hydrogen Uptake and Release in Carbon Nanotube Electrocatalysts. *Nanomaterials* 2021, *11*, 975

**DOI:** 10.3390/nano16010017

**Published:** 2025-12-22

**Authors:** Rui Lobo, Jorge Ribeiro, Filipe Inok

**Affiliations:** 1 Laboratory of Nanophysics/Nanotechnology and Energy (N2E), Center of Technology and Systems (CTS-UNINOVA), Universidade Nova de Lisboa, 2829–516 Caparica, Portugal; jhfribeiro71@gmail.com (J.R.); afbi@campus.fct.unl.pt (F.I.); 2 Department of Physics, Nova School of Science & Technology, Nova University of Lisbon, 2829–516 Caparica, Portugal

The journal retracts the article “Hydrogen Uptake and Release in Carbon Nanotube Electrocatalysts” [1] cited above.

Following publication, concerns were brought to the attention of the Editorial Office regarding the integrity of the data and redundancy between publications [1,2] that share a common corresponding author and were under evaluation at the same time.

Adhering to our standard procedure, an investigation was conducted by the Editorial Office and Editorial Board, with input from the Ethics Committee NOVA School of Science and Technology at Nova University of Lisbon, Portugal, which was unable to confirm the accuracy of the 2nd and 3rd author affiliation and origin of the study. Additionally, indications of inappropriate editing and duplication between Figure 2 and Figure 3 presented in this article [1] and Figure 3 and Figure 5 published in the later publication [2] were identified. Consequently, the Editorial Board has lost confidence in the reliability of the findings and has decided to retract this publication, as per MDPI’s retraction policy (https://www.mdpi.com/ethics#_bookmark30).

This retraction was approved by the Editor-in-Chief of the journal *Nanomaterials*.

Authors disagree with this retraction.
